# A multifactorial model of factors associated with PTSD, anxiety and depression among migrants in Spain

**DOI:** 10.3389/fpsyg.2026.1848469

**Published:** 2026-08-26

**Authors:** Helena Sainz-Elías, Iago Giné-Vázquez, Fajar Matloob Butt Butt, Wala Ayad Ahmed, Ivet Bayes-Marin, Amanda Lloret-Pineda, Marta Franch-Roca, Blanca Mellor-Marsá, Aina Gabarrell-Pascuet, Felipe Villalobos, Maria Del Carmen Alos-Belenguer, Yuelu He, Elisabeth Salomon Mallat, Lola Aparicio, Mercedes Espinal Cabeza, Óscar Álvarez Bobo, Yolanda Osorio López, Josep Maria Haro, Paula Cristóbal-Narváez

**Affiliations:** 1 Parc Sanitari Sant Joan de Déu, Institut de Recerca Sant Joan de Déu (IRSJD), Sant Boi de Llobregat, Spain; 2 Departament de Medicina, Facultat de Medicina i Ciències de la Salut, Universitat de Barcelona (UB), Barcelona, Spain; 3 San Carlos University Clinic Hospital, Madrid, Spain; 4 Department of Legal Medicine, Psychiatry and Pathology, School of Medicine, Universidad Complutense de Madrid, Madrid, Spain; 5 Department of Medicine, Universitat Internacional de Catalunya (UIC), Barcelona, Spain; 6 Instituto de Salud Carlos III, Centre for Biomedical Research on Mental Health (CIBERSAM), Madrid, Spain; 7 Parent and Family Wellness Center, Boulder, CO, United States; 8 Colegio de la Psicología de Madrid, Madrid, Spain; 9 Department of Public Health, Aarhus University, Aarhus, Denmark; 10 Barcelona Institute for Global Health (ISGlobal), Barcelona, Spain; 11 Fundació Institut Universitari per a la Recerca a l’Atenció Primària de Salut Jordi Gol i Gurina (IDIAPJGol), Barcelona, Spain; 12 Fundación Hospitalàrias, Barcelona, Spain; 13 Servei d’Atenció a la Migració en Salut Mental (SATMI), Parc Sanitari Sant Joan de Déu, Barcelona, Spain

**Keywords:** anxiety, depression, mental health, migrants, post-traumatic stress disorder (PTSD), quality of life (QoL), Spain

## Abstract

**Background:**

Migrants are at increased risk for mental health problems due to stressors before, during, and after migration. However, evidence on the factors associated with migrant mental health in Spain remains limited.

**Objectives:**

To examine how four domains– (1) sociodemographic and psychological characteristics, (2) migration-related factors, (3) social and occupational conditions, and (4) health-related quality of life factors–are associated with post-traumatic stress disorder (PTSD), anxiety, and depression among migrants.

**Methods:**

A cross-sectional survey was conducted between June 2021 and October 2022 among 509 adult migrants residing in Spain. PTSD, anxiety, and depression were assessed with the Post-Traumatic Stress Disorder Checklist for DSM-5 (PCL-5), the 7-item Generalized Anxiety Disorder Scale (GAD-7), and the Composite International Diagnostic Interview (CIDI), respectively. Multivariable logistic regression and Tobit models were used to examine associations between the four domains and mental health outcomes.

**Results:**

Women reported higher anxiety and depression, whereas younger age was associated with higher anxiety. Migrants from the Americas reported higher anxiety and depression, whereas those from Europe exhibited greater PTSD symptomatology, although this finding should be interpreted with caution given the very small European subsample. Childhood trauma was positively associated with PTSD, anxiety, and depression. Regarding migration-related factors, frequent acculturative stress and longer length of stay in Spain were associated with higher anxiety, whereas travelling alone was associated with higher depression. Within the post-migration social environment, perceived discrimination was positively associated with both PTSD and anxiety. Regarding pandemic-related factors, COVID-19-related stress was positively associated with PTSD but inversely associated with depression, whereas general stress was consistently associated with all three mental health outcomes. Lower physical quality of life was associated with higher anxiety and depression, while lower mental quality of life was associated with worse PTSD, anxiety, and depression.

**Conclusion:**

The findings support a multifactorial understanding of migrant mental health, highlighting the combined influence of pre-migration adversities, migration-related experiences and post-migration conditions. Interventions targeting modifiable factors, particularly acculturative stress, perceived discrimination, and quality of life, may contribute to improving mental health among migrant populations.

## 1 Introduction

International migration has increased substantially in recent decades, particularly toward high-income countries, reshaping their demographic composition. In 2024, there were 304 million international migrants worldwide, accounting for 3.7% of the global population, including 94.1 million residing in Europe (United Nations Department of Economic and Social Affairs, 2024). Spain reflects this broader demographic trend, with 8.9 million foreign-born individuals, representing 18.3% of the total population, according to data from the National Institute of Statistics (Instituto Nacional de Estadística, 2024). As migration continues to grow worldwide, concerns about the mental health of migrant populations have become an increasingly important public health priority. Evidence from Spain indicates that migrants–particularly refugees–experience poorer mental health than the general population (Spiritus-Beerden et al., 2021). However, despite growing interest, it remains unclear how migration-related adversities and more recent stressors, such as those arising from the COVID-19 pandemic, influence mental health outcomes (Aragona et al., 2022; Solà-Sales et al., 2021).

A growing body of evidence supports a transdiagnostic perspective on migrant mental health, with post-traumatic stress disorder (PTSD), anxiety, and depression consistently identified as the most prevalent disorders and frequently sharing common underlying risk factors (Acharya et al., 2022; Jurado et al., 2017). Although research has largely focused on refugees and asylum seekers (Bustamante et al., 2018; James et al., 2022; Nesterko et al., 2020; Sangalang et al., 2019), non-refugee migrant groups have received comparatively less attention despite facing significant psychosocial stressors throughout the migration process. According to the World Health Organization (WHO), approximately 17% of individuals exposed to outbreaks or conflicts experience mild-to-moderate mental health conditions, including anxiety, depression, and stress-related disorders (Acharya et al., 2022). These challenges have become even more pronounced in the post-pandemic context, where global health, social, and economic consequences disproportionately affected vulnerable populations, including migrants (Aragona et al., 2022; Spiritus-Beerden et al., 2021).

Migration is a complex and dynamic process characterized by multiple stressors before, during, and after displacement. The cumulative burden of these stressors increases vulnerability and may further hinder access to healthcare services (Alarcão et al., 2022). Accordingly, understanding migrant mental health requires a comprehensive perspective that accounts for the multiple factors operating across the migration process. Previous evidence (Jurado et al., 2017) has conceptualized risk and protective factors within three broad domains: (i) sociodemographic and psychological characteristics, (ii) migration-related factors, and (iii) social and occupational factors in the host country.

Within the first domain of sociodemographic and psychological characteristics, gender has emerged as one of the most robust factors, with women showing a higher risk of common mental disorders than men, particularly in contexts of socioeconomic disadvantage and gender-based violence (Alarcão et al., 2022; Borrell et al., 2010; Jurado et al., 2017; Meadows et al., 2001). Prior traumatic experiences also represent a major risk factor for poor mental health (Teodorescu et al., 2012). In addition, low self-esteem has been linked to greater psychological distress among migrants (Borrell et al., 2010), whereas higher resilience has been associated with lower levels of anxiety and depression, underscoring its protective role in coping with migration-related adversity (Gambaro et al., 2020).

The second domain comprises factors intrinsic to the migration process. Among these, acculturative stress–the psychological strain associated with adapting to a new sociocultural environment–has consistently been identified as a key migration-related factor of poorer psychological well-being (Choy et al., 2021). Other migration-related circumstances, including forced migration, migrating without close family members, uncertainty regarding the intention to remain in the host country, precarious or irregular residence status, and longer length of stay, have also been linked to poor mental health by influencing access to resources and opportunities for social adaptation to the host society (James et al., 2022; Jurado et al., 2017).

At the same time, the third domain of post-migration social and occupational conditions plays a pivotal role in shaping mental health. Socioeconomic hardship, social exclusion, and discrimination have been consistently linked to depression, anxiety, psychological distress, reduced life satisfaction, and poorer physical health (Brance et al., 2022; Pascoe and Smart Richman, 2009; Spiritus-Beerden et al., 2021). In contrast, social support has consistently been identified as a protective factor, mitigating symptoms of anxiety, depression, and stress (Doma et al., 2022). Notably, the COVID-19 pandemic has further intensified these challenges by introducing substantial socioeconomic, health and educational disruptions that disproportionately affected vulnerable populations, including migrants and individuals with pre-existing mental disorders (Acharya et al., 2022; Alarcão et al., 2022; Aragona et al., 2022; Pinzón-Espinosa et al., 2021; Spiritus-Beerden et al., 2021). By exacerbating existing social and structural inequalities, the pandemic further increased the risk of adverse mental health outcomes among populations already exposed to cumulative adversity (Alarcão et al., 2022; Aragona et al., 2022).

Building on this conceptual framework, the present study incorporates health-related quality of life (QoL) as a fourth domain, given its close association with mental health. The WHO defines QoL as individuals’ perceptions of their physical and mental health, social relationships, and environment (Bayram et al., 2007; Ghazinour et al., 2004; Nesterko et al., 2013). Consistent evidence indicates that poorer QoL is associated with higher levels of psychological distress, anxiety, and depression, although the direction of this relationship remains uncertain (Van der Boor et al., 2020). Considering QoL alongside other domains provides a broader perspective on migrants’ well-being, extending the focus beyond psychopathology to encompass social functioning, physical health, and subjective life satisfaction. This perspective is particularly relevant for migrant populations, whose mental health is shaped by complex interactions between individual, social, and structural factors (Eslam Parast and Taştekin Ouyaba, 2022; Van der Boor et al., 2020).

Taken together, despite the growing body of evidence on migrant mental health, existing research has predominantly examined isolated risk factors, with limited integration of the multiple domains that jointly shape mental health. To address this gap, the present study examines four domains–sociodemographic and psychological characteristics, migration-related factors, social and occupational conditions, and health-related QoL– in relation to PTSD, anxiety, and depression among adult migrants residing in Spain.

## 2 Materials and methods

### 2.1 Study design and sample recruitment

A cross-sectional analysis was conducted using data collected from a survey of the migrant population residing in Spain between June 2021 and October 2022. The survey was administered anonymously through the Qualtrics platform (Qualtrics, 2005) and disseminated using a non-probabilistic convenience sampling and snowball method via web-based platforms and social media channels. Eligibility criteria included adults aged 18 years or older who identified as migrants and had been residing in Spain during the study period. A total of 509 respondents participated in the survey.

The study received ethical approval from the Ethics Committee of Fundació Sant Joan de Déu, Barcelona, Spain (PIC 86-20). All participants provided informed consent electronically in accordance with the Declaration of Helsinki, by affirming agreement to participate via an online consent form.

### 2.2 Variables

#### 2.2.1 Sociodemographic and psychological variables

Descriptive analyses included sociodemographic and psychological characteristics.

Gender was categorized as women, men, or other (including transgender, agender, and gender-fluid identities). Age was recorded as a continuous variable in years. Countries of origin were grouped by continent (America, Asia, Africa, and Europe) due to the heterogeneity of countries represented in our sample and the limited number of participants from some individual nations. Civil status was classified as in a relationship or single. Education level was assessed based on the highest level of formal education completed by each participant. The variable comprised six response categories: (1) primary education, (2) secondary education, (3) high school or preparatory education, (4) technical education, (5) university higher education (e.g., bachelor’s, master’s, or equivalent), and (6) other.

Self-esteem was measured using the Rosenberg Self-Esteem Scale (RSES; Rosenberg, 1979), a 10-item self-report instrument designed to assess global self-worth and self-acceptance. Items are rated on a 4-point Likert scale, reflecting levels of agreement with positive and negative self-evaluations. Total scores range from 0 to 30, with higher scores indicating greater self-esteem and a more positive global self-concept. The scale demonstrated acceptable internal consistency (Cronbach’s α = 0.79).

Resilience was measured with the 10-item Connor-Davidson Resilience Scale (CD-RISC; Connor and Davidson, 2003), a self-report instrument designed to measure the ability to cope with adversity and adapt to stressful life events. Items are rated on a 5-point Likert scale ranging from 0 (not true at all) to 4 (true nearly all the time). Total scores are calculated as the mean of all items, with higher scores indicating greater perceived resilience. This instrument demonstrated excellent internal consistency (Cronbach’s α = 0.91). Childhood Trauma was evaluated using the Childhood Trauma Questionnaire (CTQ; Hagborg et al., 2022), a 28-item self-report instrument assessing experiences of emotional, physical, and sexual abuse, as well as emotional and physical neglect during childhood. Items were rated on a 5-point Likert scale ranging from 1 (never true) to 5 (very often true), reflecting the frequency of adverse childhood experiences. Higher scores indicate greater exposure to childhood trauma. The instrument showed excellent internal consistency (Cronbach’s α = 0.94).

#### 2.2.2 Factors related to the migration process

Migration-related factors included the type of migration, acculturative stress, intention to remain in Spain, legal residence status, European nationality, accompaniment upon arrival, and duration of residence in Spain.

Type of migration was categorized as voluntary (motivated by work, education, or quality of life) or forced (resulting from conflict, persecution, natural disasters, or threats to personal safety). Acculturative stress was assessed with the question: “*How often does the process of understanding the customs and traditions, the language, and interacting with the local people cause any kind of stress in your daily life?”* Responses were rated on a 4-point Likert scale, ranging from 1 (never) to 4 (very often), with higher scores indicating greater acculturative stress. Intention to remain in Spain was coded dichotomously as uncertain or permanent.

Legal residence status distinguished participants with permits (citizenship, temporary, permanent, and student) from those without or pending documentation. European nationality distinguished participants from EU/EEA countries from those of non-EU countries. It should be noted that continent of origin and European nationality are distinct variables in this study. Continent of origin reflects the country where each participant was born or from which they originate, whereas European nationality refers to current legal citizenship status within an EU/EAA country. Travelling companion status was assessed at arrival and categorized as alone or accompanied by family members, friends, or other individuals. Duration of residence in Spain was measured continuously in years and months.

#### 2.2.3 Factors related to the social and occupational environment in the host country

Factors related to the social and occupational environment in the host country included the employment contract, perceived discrimination, social support, feelings of inferiority, and stressful life events.

Employment-related variables included paid employment experience in Spain, occupational position during the last 12 months, and current employment contract status. Participants were asked whether they had ever engaged in paid employment in Spain (yes/no). Occupational position during the previous 12 months was categorized into broad occupational groups, including elementary and low-skilled service occupations, manual and technical occupations, sales and small business occupations, professional occupations, and managerial positions. The current employment contract was operationalized as a presence indicator and recorded dichotomously (yes/no).

Perceived discrimination was measured using the 5-item Everyday Discrimination Scale (EDS; Lawrence et al., 2022), a 5-item self-report instrument assessing the frequency of perceived discrimination in daily life. Participants rated five items about their perceptions of discrimination: (e.g., *“Are you treated with less courtesy than other people?”*) on a 6-point Likert scale ranging from 0 (never) to 5 (almost every day). In this study, total EDS scores were calculated by summing responses across the five items, with higher scores indicating greater perceived discrimination. Internal consistency was good (Cronbach’s α = 0.81).

Social support was assessed with the 6-item MOS Social Support Survey - Short Form (MOS-SSS-6; Dao-Tran et al., 2023), a brief self-report instrument that measures perceived functional social support, including emotional, informational, and instrumental support. Each item was rated on a 5-point Likert scale (0–4) to reflect perceived availability of supportive resources in daily life. The overall score was computed as the mean of all items, with higher values indicating greater perceived social support. Internal consistency was excellent (Cronbach’s α = 0.91).

Feeling inferiority was assessed by using the question: “Since you arrived in Spain, have you ever felt inferior to others because of your ethnicity, religion, or skin color?. The item captures the subjective perceptions of social comparison, marginalization and self-perceived social status related to migration and minority identity. Responses were dichotomized as yes or no.

Stressful life events were measured using the List of Threatening Experiences (LTE), a 20-item scale assessing major life stressors experienced during the previous 6 months (Brugha and Cragg, 1990). Each event was recorded dichotomously (yes/no). Items include serious illnesses or injury (self or close relative), death of a close family member or friend, relationship breakdown, financial hardship, and job loss. Total scores ranged from 0 to 20, with higher scores indicating greater exposure to stressors. Internal consistency was acceptable (Cronbach’s α = 0.78).

##### 2.2.3.1 Pandemic context

Within the context of the COVID-19 pandemic, several items were incorporated into the survey to assess its impact on participants’ mental health.

COVID-19 diagnosis was assessed by asking whether participants had been diagnosed by a physician or hospitalized due to COVID-19-related symptoms. Responses indicated whether participants had received a formal diagnosis or required medical attention for COVID-19-related health issues.

An adapted version of the Peri Life Events Scale (Dohrenwend et al., 1978) was employed to assess stressors experienced during the pandemic. In the present analysis, the 13 items of the scale were divided into two subscales to differentiate sources of stress: COVID-related stress and general stress. The COVID-related stress subscale included five items referring specifically to pandemic-related concerns, such as worry about being infected with coronavirus, worry that loved ones might become infected, the death of a loved one due to coronavirus, loss of job or income as a result of the pandemic, and exposure to negative or alarming messages about coronavirus in the media (Cronbach’s α = 0.81). The general stress subscale comprised eight items assessing broader life stressors unrelated to the pandemic, including concerns about one’s own health, the health of loved ones, romantic relationships, family relationships, problems affecting loved ones, financial situation, difficulties getting along with coworkers, and life in general (Cronbach’s α = 0.88). For the full 13-item scale, internal consistency was high (Cronbach’s α = 0.91).

In addition, residency status during the pandemic was recorded by asking participants whether they had lived in Spain continuously since March 2020, up to the time of completing the survey. Responses included: *”Yes, throughout the entire period”, “Yes, in Spain but also in other countries”, and “No, I have been living outside of Spain”.*

Finally, to account for potential temporal variability across the pandemic data-collection period, a continuous variable labeled time since study onset was included in all regression models. This variable was operationalized as the number of days elapsed since the first recorded survey response (10 June 2021) and was incorporated to adjust for potential changes in pandemic-related conditions and contextual factors throughout the recruitment period (the effective recruitment and survey completion period included in the analyses ranged between June 2021 and October 2022).

#### 2.2.4 Health-related quality of life (QoL)

Health-related QoL was assessed using the Short Form Health Survey (SF-12; Ware and Keller, 1998), a widely used general health questionnaire comprising 12 items covering eight dimensions: general health perception (1 question), physical health (2 questions), limited physical role function (2 questions), physical pain (1 question), vitality (1 question), mental health (2 questions), limited emotional role function (2 questions), and social functioning (1 question). These domains were aggregated into two composite scores – the Mental Component Score (MCS-12) and the Physical Component Score (PCS-12) – for subsequent analyses (Cronbach’s α = 0.82).

#### 2.2.5 Mental health outcomes: post-traumatic stress disorder (PTSD), anxiety and depression

Mental health outcomes were assessed using validated measures of post-traumatic stress disorder (PTSD), anxiety and depression.

Post-traumatic stress disorder (PTSD) was measured using a 4-item self-report questionnaire, the PTSD Checklist for DSM-5 (PCL-5), derived from the DSM-5 diagnostic criteria (Zuromski et al., 2019). It measures perceived distress over the past month on a Likert Scale ranging from 0 (not at all) to 4 (extremely). The items included re-experiencing of stressful events, avoidance of reminders of the stressor, numbing of general responsiveness, and increased arousal. The total score ranged from 0 to 16, with higher scores reflecting greater PTSD symptom severity. The scale demonstrated high internal consistency (Cronbach’s alpha α = 0.85).

Anxiety was measured with the 7-item Generalized Anxiety Disorder Scale (GAD-7; Spitzer et al., 2006), a validated screening instrument for generalized anxiety disorder and other anxiety conditions. Participants rated the frequency of symptoms over the past 2 weeks on a 4-point Likert scale. Symptoms included: feeling nervous, anxious or on edge; difficulty controlling worry; excessive worrying; trouble relaxing; restlessness; becoming easily annoyed or irritable; and feeling as though something awful might happen. Total scores ranged from 0 to 21, with higher scores indicating greater symptom severity. The scale demonstrated strong internal consistency, with Cronbach’s α = 0.90 in our sample.

Depression was evaluated using the Composite International Diagnostic Interview (CIDI; Haro et al., 2006), a structured psychiatric questionnaire developed internationally from the Diagnostic Interview Schedule and sponsored by the WHO. The CIDI allows for the assessment of major depressive symptoms, including their presence, duration, periodicity, and intensity, through algorithm-based responses that yield a dichotomous classification (Yes/No).

### 2.3 Statistical analyses

All continuous variables were expressed as means and standard deviations (SD), whereas categorical variables were presented as frequencies and percentages.

To examine associations between mental health outcomes and the described predictors, multivariable Tobit regression models were employed for the PTSD and anxiety measures, as this approach is appropriate for censored dependent variables such as GAD-7 and PTSD scores. To evaluate the association between predictors and depression, a binary logistic regression model was fitted based on the dichotomous classification defined by the CIDI algorithm.

The covariates included in the final multivariable models were: gender, age, continent of origin, civil status, self-esteem, resilience, and childhood trauma as sociodemographic and psychological variables; type of migration, acculturative stress, intention to remain in Spain, residence status, current nationality, travelling companion status, and length of stay in Spain as migration-related variables; perceived discrimination, feeling of inferiority, social support, stressful life events, and employment contract as social and occupational environment variables; residence during the COVID-19 pandemic, COVID-19 diagnosis, COVID-related stress, general stress, and time since study onset as pandemic-related variables; and SF-12 physical and mental component scores as health-related QoL. No missing data were present in the variables included in the regression analyses; therefore, no additional missing-data handling procedures were required. The effective sample size for the main regression models was *n* = 509.

Multicollinearity was assessed using generalized variance inflation factors (GVIFs; Supplementary Table 1). Across the main models, all transformed GVIF values (GVIF^[1/(2 × Df)]) were low and below conventional thresholds for concern, ranging from approximately 1.03 to 1.43, indicating no evidence of problematic multicollinearity among the predictors. Estimates of association were reported as regression coefficients for the Tobit models and odds ratios for the logistic regression model, together with corresponding standard errors and *p*-values. Statistical significance was defined as a two-sided *p-*value < 0.05. In addition, to assess the robustness of the Europe-related PTSD finding, a sensitivity analysis was additionally conducted in which continent of origin was recoded as a binary variable (Europe vs. non-Europe), the current nationality covariate was removed, and robust sandwich standard errors were computed (Supplementary Table 2).

All the analyses were conducted using R software (version 4.4.2) (R Core Team, 2024), with the R packages AER (version 1.2-14) (Kleiber and Zeileis, 2008) for the Tobit regression models and ltm (version 1.2-0) (Rizopoulos, 2006) for estimating Cronbach’s α coefficients, car (version 3.1-5) (Fox and Weisberg, 2019) for computing generalized variance inflation factors (GVIFs), and sandwich (version 3.1-1) (Zeileis, 2004) for computing robust standard errors in the sensitivity analysis.

## 3 Results

### 3.1 Description of the overall sample

The characteristics of the study sample are presented in Table 1. A total of 509 participants were included.

**TABLE 1:** Main descriptive statistics of sociodemographic, psychological, migration-related, social environment, and health variables among migrant participants (*N* = 509).

Variables		Total sample (*n* = 509)
Sociodemographic and psychological characteristics		
Gender, *n* (%)	Men	186 (36.5%)
	Women	319 (62.7%)
	Other (transgender, agender, gender fluid)	4 (0.8%)
Age, mean (SD)		34.71 (10.27)
Continent of origin	Africa	79 (15.5%)
	America	293 (57.6%)
	Asia	131 (25.7%)
	Europe	6 (1.2%)
Civil status	Single	250 (49.1%)
	In a relationship	259 (50.9%)
Educational level	Primary education	37 (7.3%)
	Secondary education	125 (24.6%)
	High school / preparatory education	37 (7.3%)
	Technical or vocational higher education	97 (19.1%)
	University education	189 (37.1%)
	Other	24 (4.7%)
Self-esteem (RSES; 0–30), mean (SD)		21.31 (4.65)
Resilience (CD-RISC 10; 0–4), mean (SD)		2.46 (0.96)
Childhood trauma (CTQ; 25–125), mean (SD)		42.92 (17.26)
Migration-related factors		
Type of migration	Voluntary	371 (72.9)
	Forced	138 (27.1)
Acculturative **s**tress	Never	122 (24%)
	Occasionally	327 (64.2%)
	Often	45 (8.8%)
	Very often	15 (2.9%)
Intention to remain in Spain	Uncertain	238 (46.8%)
	Intention to stay	271 (53.2%)
Residence status	Undocumented or in process	184 (36.1%)
	Residence permit	325 (63.9%)
Nationality	European	46 (9%)
	Non-European	463 (91%)
Travelling companion status	Accompanied	244 (47.9%)
	Alone	265 (52.1%)
Length of stay in Spain, mean (SD)		5.61 (6.70)
Social and occupational environment factors		
Perceived discrimination (EDS; 0–25), mean (SD)		5.57 (4.44)
Feeling of inferiority	Yes	271 (53.2%)
	No	238 (46.8%)
Social support (MOS-SSS-6; 0–4), mean (SD)		2.23 (1.07)
Stressful life events (LTE; 0–20), mean (SD)		4.16 (3.37)
Ever engaged in paid employment in Spain	Yes	338 (66.4)
	No	171 (33.6)
Main occupational category over the past 12 months (ever-employed participants only)	Elementary occupations and low-skilled service work	135 (39.9)
	Unskilled manual and service occupations	48 (14.2)
	Semi-skilled technical and manual occupations	24 (7.1)
	Skilled trades, artisanal, and self-employed manual occupations	9 (2.7)
	Sales and small business occupations	33 (9.8)
	Technical and associate professional occupations	24 (7.1)
	Lower professional and supervisory occupations	30 (8.9)
	Administrative and intermediate managerial occupations	15 (4.4)
	Higher professional and executive occupations	20 (5.9)
Current employment contract	Yes	124 (24.4%)
	No	385 (75.6%)
Residence during the COVID-19 pandemic	Yes, during the entire period	419 (82.3%)
	Yes, but also in other countries	31 (6.1%)
	No	59 (11.6%)
COVID-19 diagnosis	Yes	125 (24.6%)
	Yes and hospitalization	10 (2%)
	No	374 (73.5%)
COVID-related stress (Adapted Peri Life Events Scale; 0–20)	9.07 (5.24)	
General **s**tress (Adapted Peri Life Events Scale; 0–32)	15.24 (7.55)	
Time since study onset, mean (SD)	129 (94.2)	
Health-related quality of life (SF-12)		
Mental component score (MCS-12; 0–100)		44.14 (10.90)
Physical component score (PCS-12; 0–100)		48.92 (9.10)
Mental health		
Post-traumatic stress disorder (PTSD; PCL-5; 0–16), mean (SD)		4.60 (3.76)
Anxiety (GAD-7); 0–21, mean (SD)		7.33 (5.49)
Depression (CIDI)	Yes	120 (23.6%)
	No	389 (76.4%)

Values are presented as mean (SD) for continuous variables and as percentages for categorical variables. RSES, Rosenberg Self-Esteem Scale; CD-RISC-10, Connor–Davidson Resilience Scale; CTQ, Childhood Trauma Questionnaire; EDS, Everyday Discrimination Scale; MOS-SSS-6, Medical Outcomes Study Social Support Survey – Short Form; LTE, List of Threatening Experiences; SF-12, 12-Item Short Form Health Survey; MCS-12, Mental Component Summary (SF-12); PCS-12, Physical Component Summary (SF-12); PTSD, Post-Traumatic Stress Disorder; PCL-5, PTSD Checklist for DSM-5; GAD-7, Generalized Anxiety Disorder Scale (7-item); CIDI, Composite International Diagnostic Interview. The table summarizes participants according to continent of origin. For completeness, the distribution by country of origin is provided here: Africa: Algeria (1.2%), Cape Verde (0.2%), Cameroon (0.4%), Côte d’Ivoire (0.2%), Egypt (0.2%), Gambia (0.4%), Guinea (0.2%), Equatorial Guinea (0.4%), Kenya (0.2%), Mali (0.4%), Morocco (10.8%), Niger (0.4%), and Senegal (0.6%); America: Argentina (5.7%), Bolivia (1.4%), Brazil (1.2%), Chile (2.6%), Colombia (10.4%), Costa Rica (0.2%), Cuba (3.1%), Ecuador (1.8%), El Salvador (1.2%), Guatemala (0.4%), Haiti (0.4%), Honduras (4.9%), Mexico (1.0%), Nicaragua (0.8%), Peru (7.1%), the Dominican Republic (0.2%), Uruguay (0.4%), and Venezuela (14.9%); Asia: China (9.6%), India (0.2%), Pakistan (15.7%), and Singapore (0.2%); Europe: Austria (0.2%), Italy (0.2%), Romania (0.2%), the United Kingdom (0.2%), and Ukraine (0.4%).

Regarding sociodemographic and psychological characteristics, most participants identified as women (62.7%), followed by men (36.5%), and other gender identities (0.8%; including transgender, agender, or gender-fluid identities). The mean age was 34.71 years (*SD* = 10.27). Owing to the limited representation of several nationalities, statistical analyses were conducted according to continent of origin. Participants originated from Africa (15.5%), America (57.6%), Asia (25.7%), and Europe (1.2%).

Approximately half of the participants were in a relationship (50.9%). University education was the most frequently reported educational level (37.1%), followed by secondary and technical/vocational education, whereas primary and high school/preparatory education were the least common (both 7.3%). Psychological characteristics in our sample indicated moderate levels of self-esteem (*M* = 21.31, SD = 4.65) and resilience (*M* = 2.46, SD = 0.96), alongside relatively moderate childhood trauma exposure, suggesting heterogeneity in early adverse experiences within the sample (*M* = 42.92, SD = 17.26).

Migration-related factors indicated that most participants had migrated voluntarily (72.9%), whereas the remainder reported forced migration. Acculturative stress was most frequently reported as occasional (64.2%). Slightly more than half of the participants (53.2%) intended to stay permanently, while the remainder were uncertain. Most participants held a residence permit (63.9%), while over one-third were undocumented or in the process of regularization. Only a small proportion held European nationality (9%), with the vast majority (91.0%) being non-European. As described in the section “2 Materials and methods,” the proportion of participants originating from Europe (1.2%, *n* = 6) differed from the proportion holding European nationality (9.0%, *n* = 46), as country of origin and legal citizenship represent distinct variables. In terms of travelling companion status, just over half of participants migrated alone (52.1%), while the rest migrated accompanied by a travelling companion. The mean duration of residence in Spain was 5.61 years (SD = 6.70).

Concerning social and occupational factors, participants reported low-to-moderate levels of perceived discrimination (*M* = 5.57, SD = 4.44), and 53.2% reported feelings of inferiority. Perceived social support was moderate (*M* = 2.23, SD = 1.07), and stressful life events were low-to-moderate (*M* = 4.16, SD = 3.37). Overall, 66.4% reported having engaged in paid employment in Spain, whereas 33.6% had never worked in Spain. Among those with previous employment experience, the most common occupational category during the last 12 months was elementary occupations and low-skilled service work (39.9%), followed by unskilled manual and service occupations (14.2%). Only 24.4% of participants reported having a current employment contract.

Pandemic-related factors indicated that 82.3% of participants lived continuously in Spain during the pandemic, 6.1% lived between Spain and other countries, and 11.6% resided outside Spain. A total of 24.6 % reported a COVID-19 diagnosis, and 2.0% required hospitalization. COVID-related stress (*M* = 9.07, SD = 5.24) and general stress (*M* = 15.24, SD = 7.55) indicated moderate exposure to stressors during the pandemic.

Finally, health-related QoL scores indicated slightly below-average mental health (*M* = 44.14, SD = 10.90) and near-average physical functioning (*M* = 48.92, SD = 9.10). Mental health outcomes showed mild to moderate symptom levels for PTSD (*M* = 4.60, SD = 3.76) and anxiety (*M* = 7.33, SD = 5.49), while 23.6% of participants met criteria for depression.

### 3.2 Risk and protective factors on PTSD, anxiety and depression

Regression analyses identified several factors associated with mental health outcomes (PTSD, anxiety and depression) (see Table 2).

**TABLE 2:** Multivariable regression analyses of four domains associated with PTSD, anxiety, and depression among migrant participants (**N** = 509).

Mental health outcomes		PTSD			Anxiety			Depression		
Factor domains		B (SE)	(CI95%)	*P*-value	B (SE)	(CI95%)	*P*-value	OR (SE)	(CI95%)	*P*-value
Sociodemographic and psychological characteristics										
Gender	Male	Ref.			Ref.			Ref.		
	Female	0.17 (0.33)	(−0.49, 0.82)	0.621	**1.02 (0.43)**	**(0.17, 1.86)**	**0.018**	**2.59 (0.84)**	**(1.39, 5.00)**	**0.003**
	Other (transgender, agender, gender fluid)	**−4.35 (1.70)**	**(−7.69, −1.02)**	**0.011**	0.44 (2.31)	(−4.09, 4.97)	0.849	0.44 (0.58)	(0.03, 5.78)	0.531
Age		−0.01 (0.02)	(−0.05, 0.02)	0.413	**−0.06 (0.02)**	**(−0.10, −0.02)**	**0.004**	0.99 (0.02)	(0.96, 1.02)	0.428
Continent of origin	Africa	Ref.			Ref.			Ref.		
	America	0.66 (0.50)	(−0.31, 1.64)	0.182	**1.85 (0.64)**	**(0.60, 3.10)**	**0.004**	**3.39 (1.67)**	**(1.33, 9.31)**	**0.013**
	Asia	0.28 (0.52)	(−0.74, 1.30)	0.589	−0.66 (0.67)	(−1.97, 0.65)	0.322	1.66 (0.83)	(0.63, 4.55)	0.313
	Europe	**3.35 (1.44)**	**(0.53, 6.16)**	**0.020**	0.57 (1.92)	(−3.19, 4.33)	0.766	5.80 (7.159)	(0.39, 58.14)	0.154
Civil status	In a relationship	Ref.			Ref.			Ref.		
	Single	0.39 (0.33)	(−0.26, 1.04)	0.245	−0.28 (0.43)	(−1.12, 0.56)	0.515	0.79 (0.23)	(0.44, 1.41)	0.427
Self-esteem		−0.02 (0.04)	(−0.10, 0.06)	0.602	−0.00 (0.05)	(−0.10, 0.10)	0.955	1.01 (0.04)	(0.94, 1.08)	0.828
Resilience		0.02 (0.17)	(−0.31, 0.35)	0.904	0.10 (0.21)	(−0.32, 0.52)	0.632	1.27 (0.20)	(0.94, 1.73)	0.132
Childhood trauma		**0.04 (0.01)**	**(0.02, 0.06)**	**<0.001**	**0.03 (0.01)**	**(0.00, 0.05)**	**0.040**	**1.02 (0.01)**	**(1.00, 1.03)**	**0.049**
Migration-related factors												
Type of migration	Voluntary	Ref.			Ref.			Ref.		
	Forced	0.02 (0.38)	(−0.72, 0.76)	0.963	0.17 (0.49)	(−0.79, 1.13)	0.733	1.16 (0.40)	(0.59, 2.26)	0.662
Acculturative stress	Never	Ref.			Ref.			Ref.		
	Occasionally	0.57 (0.39)	(−0.20, 1.33)	0.146	0.18 (0.50)	(−0.81, 1.16)	0.724	0.64 (0.23)	(0.32, 1.30)	0.212
	Often	0.60 (0.62)	(−0.62, 1.81)	0.337	0.36 (0.80)	(−1.20, 1.92)	0.653	0.33 (0.19)	(0.10, 1.00)	0.055+
	Very often	1.05 (0.97)	(−0.84, 2.94)	0.278	**4.48 (1.29)**	**(1.96, 7.00)**	**0.001**	3.55 (3.06)	(0.66, 19.63)	0.141
Intention to remain in Spain	Uncertain	Ref.			Ref.			Ref.		
	Intention to stay	−0.17 (0.32)	(−0.79, 0.46)	0.599	0.30 (0.41)	(−0.50, 1.11)	0.459	1.21 (0.34)	(0.70, 2.11)	0.496
Residence status	Undocumented or in process	Ref.			Ref.			Ref.		
	Residence permit	0.24 (0.39)	(−0.52, 1.00)	0.532	−0.27 (0.50)	(−1.25, 0.72)	0.596	0.48 (0.27)	(0.40, 1.53)	0.467
Current nationality	European	Ref.						Ref.		
	Non-European	0.64 (0.56)	(−0.45, 1.73)	0.252	−0.85 (0.71)	(−2.25, 0.54)	0.231	1.23 (0.59)	(0.49, 3.24)	0.671
Travelling companion status	Accompanied	Ref.			Ref.			Ref.		
	Alone	0.44 (0.33)	(−0.20, 1.08)	0.174	−0.13 (0.42)	(−0.95, 0.70)	0.762	**1.81 (0.54)**	**(1.01, 3.26)**	**0.047**
Length of stay in Spain		0.01 (0.03)	(−0.04, 0.07)	0.703	**0.09 (0.04)**	**(0.01, 0.16)**	**0.020**	1.00 (0.03)	(0.95, 1.06)	0.887
Social and occupational environment factors												
Perceived discrimination		**0.15 (0.04)**	**(0.07, 0.24)**	**0.001**	**0.13 (0.06)**	**(0.02, 0.24)**	**0.020**	1.00 (0.04)	(0.93, 1.08)	0.910
Feeling of inferiority	No	Ref.			Ref.			Ref.		
	Yes	−0.24 (0.34)	(−0.92, 0.43)	0.480	0.44 (0.44)	(−0.43, 1.31)	0.321	1.02 (0.32)	(0.56, 1.89)	0.939
Social support		0.13 (0.15)	(−0.17, 0.42)	0.412	−0.12 (0.20)	(−0.51, 0.27)	0.542	0.87 (0.12)	(0.66, 1.15)	0.336
Stressful life events		0.09 (0.05)	(−0.01, 0.19)	0.062+	0.04 (0.07)	(−0.09, 0.17)	0.570	1.02 (0.04)	(0.94, 1.11)	0.609
Current employment contract	No	Ref.			Ref.			Ref.		
	Yes	−0.44 (0.39)	(−1.12, 0.33)	0.265	−0.16 (0.51)	(−1.16, 0.84)	0.752	1.13 (0.41)	(0.55, 2.30)	0.744
Residence during the COVID-19 pandemic	Yes, during the entire period	Ref.			Ref.			Ref.		
	Yes, but also in other countries	0.20 (0.64)	(−1.06, 1.45)	0.759	1.39 (0.84)	(−0.26, 3.05)	0.099+	1.02 (0.58)	(0.31, 3.00)	0.968
	No	−0.36 (0.53)	(−1.39, 0.68)	0.501	0.74 (0.67)	(−0.58, 2.05)	0.271	0.50 (0.25)	(0.18, 1.28)	0.162
COVID-19 diagnosis	Yes	Ref.			Ref.			Ref.		
	Yes and hospitalization	−0.11 (1.08)	(−2.23, 2.01)	0.918	1.52 (1.41)	(−1.24, 4.28)	0.280	0.86 (0.77)	(0.12, 4.50)	0.870
	No	0.19 (0.37)	(−0.53, 0.90)	0.609	−0.35 (0.47)	(−1.27, 0.58)	0.462	0.72 (0.24)	(0.38, 1.37)	0.315
COVID-related stress		**0.17 (0.04)**	**(0.09, 0.25)**	**<0.001**	0.07 (0.05)	(−0.03, 0.17)	0.197	**0.91 (0.03)**	**(0.84, 0.97)**	**0.005**
General stress		**0.09 (0.03)**	**(0.04, 0.15)**	**0.001**	**0.09 (0.04)**	**(0.02, 0.16)**	**0.014**	**1.07 (0.03)**	**(1.02, 1.13)**	**0.007**
Time since study onset		0.00 (0.00)	(−0.00–0.01)	0.380	−0.00 (0.00)	(−0.01–0.00)	0.309	1.00 (0.00)	(1.00, 1.01)	0.222
Health-related quality of life												
Mental component score		**−0.10 (0.02)**	**(−0.13, −0.06)**	**<0.001**	**−0.27 (0.02)**	**(−0.32, −0.23)**	**<0.001**	**0.89 (0.02)**	**(0.86, 0.92)**	**<0.001**
Physical component score		−0.03 (0.02)	(−0.06, 0.01)	0.113	**−0.13 (0.02)**	**(−0.18, −0.09)**	**<0.001**	**0.95 (0.02)**	**(0.92, 0.98)**	**0.001**
Log-likelihood (Df)		-1161 (35)			-1300 (35)			–		
Wald statistic (Df)		390.1 (33)			554.0 (33)			–		
*P*-value		<2.22 × 10^−16^			<2.22 × 10^−16^			–		
AIC		–			–			456.06		

Bold values indicate statistically significant results (*p* < 0.05).

In terms of sociodemographic and psychological characteristics, being a woman was associated with higher anxiety (β = 1.02, SE = 0.43, *p* = 0.018) and depression (OR = 2.59, 95% CI: 1.39, 5.00, *p* = 0.003). Age showed a significant negative association with anxiety levels (β = –0.06, SE = 0.02, *p* = 0.004). Compared to participants from Africa (reference category), those from Europe exhibited higher PTSD scores (β = 3.35, SE = 1.44, *p* = 0.020). A sensitivity analysis (Supplementary Table 2) yielded consistent results (β = 2.73, SE = 1.21, *p* = 0.024), despite the limited subgroup size (*n* = 6). As mentioned in the section “2 Materials and Mmethods,” this analysis recoded the continent of origin as a binary variable (Europe vs. non-Europe, removed the current nationality covariate, and computed robust sandwich standard errors. In contrast, participants from America showed higher anxiety levels (β = 1.85, SE = 0.64, *p* = 0.004) and higher depression levels (OR = 3.39, 95% CI: 1.33, 9.31, *p* = 0.013). Childhood trauma was positively associated with PTSD (β = 0.04, SE = 0.01, *p* < 0.001), anxiety (β = 0.03, SE = 0.01, *p* = 0.040) and depression (OR = 1.02, 95% CI: 1.00, 1.03, *p* = 0.049).

Regarding migration-related factors, participants who reported experiencing acculturative stress very often exhibited significantly higher levels of anxiety compared to those who reported never experiencing such stress (β = 4.48, SE = 1.29, *p* = 0.001). In addition, compared with participants travelling accompanied, those travelling alone showed significantly higher odds of depression (OR = 1.81, 95% CI: 1.01, 3.26, *p* = 0.047). Longer length of stay in Spain was significantly associated with higher anxiety levels (β = 0.09, SE = 0.04, *p* = 0.020).

Within the domain of social and occupational environment factors in the host country, perceived discrimination was positively associated with PTSD scores (β = 0.15; SE = 0.04, *p* = 0.001) and with anxiety (β = 0.13; SE = 0.06, *p* = 0.020). In terms of pandemic-related factors, COVID-related stress was positively associated with PTSD (β = 0.17, SE = 0.04, *p* < 0.001) and negatively associated with depression (OR = 0.91, 95% CI: 0.84, 0.97, *p* = 0.005). However, general stress was positively associated with PTSD (β = 0.09, SE = 0.03, *p* = 0.001), anxiety (β = 0.09, SE = 0.04, *p* = 0.014) and depression (OR = 1.07, 95% CI: 1.02, 1.13, *p* = 0.007).

Finally, in relation to health-related QoL, scores on the mental component (MCS-12) were negatively associated with PTSD (β = –0.10, SE = 0.02, *p* < 0.001), anxiety (β = –0.27, SE = 0.02, *p* < 0.001), and depression (OR = 0.89, 95% CI: 0.86, 0.92, *p* < 0.001), indicating a strong association between poorer mental QoL and worse mental health outcomes overall. Similarly, lower scores on the physical component (PCS-12) were negatively associated with anxiety (β = –0.13, SE = 0.02, *p* < 0.001) and depression (OR = 0.95, 95% CI: 0.92, 0.98, *p* = 0.001), indicating that poorer physical QoL was associated with higher levels of anxiety and depression.

## 4 Discussion

The present study advances understanding of the factors associated with mental health among migrants living in Spain. Consistent with previous research in migrant populations (Jurado et al., 2017), the findings support a multifactorial perspective, highlighting the interplay of sociodemographic, psychological, migration-related, and post-migration social and occupational factors, together with health-related QoL in relation to symptoms of PTSD, anxiety, and depression. To facilitate interpretation, the discussion is organized around these four domains.

### 4.1 Sociodemographic and psychological characteristics

Migrant women showed higher levels of depression and anxiety than men, consistent with prior evidence linking these disparities to intersecting structural and interpersonal factors –such as gender roles, racism, socioeconomic disadvantage and victimization –which reinforce inequalities and limit access to protective resources and opportunities (Dalgard and Thapa, 2007; Bhugra and Gupta, 2010; Pavón Mayoral, 2014). No similar pattern was observed among participants with diverse gender identities (transgender, agender, or gender-fluid), who unexpectedly reported lower PTSD symptoms and no significant differences in depression or anxiety, likely due to their low representation in our sample (0.8% of the total sample). Nonetheless, previous research has shown that sexual and gender minorities face elevated risks of PTSD, depression, and anxiety due to discrimination, violence, and social exclusion (Lasowski et al., 2023).

Age was inversely associated with anxiety, indicating greater vulnerability among younger migrants. This finding is consistent with evidence suggesting that migration at an earlier age or bicultural upbringing may increase psychological distress through identity conflict, acculturative stress, and discrimination (Jurado et al., 2017; Lerias et al., 2025). Previous research has also suggested that the relationship between age at migration and common mental disorders is less well established and may vary according to other migration-related factors, including length of residence (Straiton et al., 2024).

Although the representation of participants by continent in our sample was uneven, several associations emerged. Migrants from America–mainly from Central and South America–exhibited higher levels of anxiety and depression compared to African migrants, while European participants showed more pronounced symptoms of PTSD. This finding should be interpreted with particular caution because the European subgroup was extremely small (*n* = 6). Although a sensitivity analysis yielded a similar result, it should not be overinterpreted. These results are consistent with previous studies conducted in migrant populations in Spain, particularly in contexts of socioeconomic disadvantage (Gómez-Salgado et al., 2024; Paloma et al., 2025). Some participants in this subgroup were from Ukraine, which may provide additional contextual background for the elevated PTSD symptoms observed (Karstoft et al., 2024). In contrast, prior research has often reported higher rates of PTSD and depression among African migrants, especially within refugee populations (James et al., 2022). This discrepancy reinforces the importance of interpreting mental health disparities within the broader context of migration experiences, including the reason for migration, the destination country and the length of stay (Straiton et al., 2024), rather than attributing them solely to country of origin.

As expected, childhood trauma was positively associated with PTSD, anxiety and depression, consistent with extensive evidence linking adverse childhood experiences to poorer mental health outcomes (Gambaro et al., 2020; Gleeson et al., 2020; Tinghög et al., 2017). Notably, it should be noted that several factors discussed in this section–including gender, age, and childhood trauma–are well-established correlates of mental health in the general population. Although these associations were also observed in the present study, the absence of a non-migrant comparison group precludes conclusions about whether they operate differently or more strongly among migrants. Instead, the findings indicate that these broadly recognized correlates remain relevant within this migrant sample and may interact with migration-related factors, an issue that future longitudinal and comparative studies should examine further.

### 4.2 Factors related to the migration process

Higher acculturative stress was positively associated with anxiety, consistent with previous research across North America, Europe and Latin America (Urzúa et al., 2019). A substantial body of evidence has linked acculturative stress to a range of adverse mental health outcomes, particularly anxiety-related and psychosomatic symptoms (Bekteshi and van Hook, 2015). Similarly, Kartal and Kiropoulos (2016) reported that acculturative stress remained associated with anxiety after accounting for pre-migration traumatic exposure, whereas no significant association was observed with depressive symptoms. Although previous studies have also linked acculturative stress to broader mental health symptoms (Baeza-Rivera et al., 2022), our findings suggest that anxiety-related outcomes may be particularly sensitive to its effects, further supporting the role of acculturative stress as a key psychosocial factor in migrant mental health (Choy et al., 2021).

Additionally, migrating alone to Spain was significantly associated with depression. This finding is consistent with previous research indicating that the absence of family or social support during the migration process may increase emotional vulnerability among migrants. Similarly, Jurado et al. (2017) reported that limited social support and separation from close networks may contribute to feelings of loneliness and difficulties adapting to the host country, thereby increasing the risk of depression.

Contrary to previous literature suggesting that migration-related factors, such as forced migration or irregular residence status, may increase vulnerability to adverse mental health outcomes (Jurado et al., 2017), these variables were not significantly associated with the outcomes in the present study. Several explanations may account for these findings. First, the limited variability of some variables may have reduced statistical power. For example, the majority of participants held a residence permit (63.9%), limiting comparisons with undocumented participants. Second, measurement limitations should be considered. A single binary item for residence status may not capture the full spectrum of administrative precarity (e.g., differences between temporary and permanent permits or uncertainty regarding permit renewal), potentially attenuating any association. Third, the heterogeneity of experiences within broad categories such as forced migration, which encompasses conflict-related displacement, persecution, and natural disasters, may have diluted associations by grouping together diverse experiences. Finally, it is plausible that post-migration factors such as discrimination and stress partially mediate or moderate the relationship between migration characteristics and mental health, making direct associations with migration type and residence status harder to detect in a cross-sectional multivariable model that simultaneously adjusts for these variables (Patel et al., 2017).

### 4.3 Social and occupational environment in the host country

A positive association was found between perceived discrimination and symptoms of PTSD and anxiety, consistent with evidence identifying discrimination as a key factor associated with mental health among migrants (Brance et al., 2022). Numerous studies have shown that experiences of discrimination and social rejection in the host country may act as major psychosocial stressors, closely associated with higher levels of psychopathology–and even with impaired physical health (Pascoe and Smart Richman, 2009).

In relation to pandemic-related factors, our findings also revealed that general stress was significantly associated with PTSD, depression and anxiety. This is consistent with previous research showing that migrants are exposed to multiple stressors that increase their vulnerability to poor mental health outcomes (Sidamon-Eristoff et al., 2022). These findings also align with systematic reviews demonstrating that migration-related stress–together with trauma exposure, pre-existing vulnerabilities and adverse post-migration conditions–contributes to a higher risk of PTSD, anxiety, and depression (Bustamante et al., 2018). By contrast, stress associated with the COVID-19 pandemic was significantly associated with PTSD, but showed inverse and non-significant associations with depressive and anxiety symptoms, respectively. This pattern suggests that pandemic-related stress, as captured in this study, may reflect a more situational and adaptive response rather than a chronic form of stress. Specifically, the COVID-related stress subscale –, which included concerns such as fear of infection, worry about loved ones, bereavement, economic loss, and exposure to alarming media–captured immediate and threat-oriented stressors. These stressors may be more closely linked with trauma-related responses, which could explain their specific association with PTSD.

In contrast, depression is typically linked to more persistent and generalized stressors, as reflected in the general stress measure. The lack of association with anxiety may indicate that, over time, individuals adapted to pandemic-related stressors, reducing their impact on anxiety symptoms. This interpretation is consistent with the timing of data collection (2021–2022), when pandemic’s initial acute phase had evolved into a more prolonged period of adjustment. During this stage, the cumulative effects of prolonged uncertainty may have contributed to longer-term emotional exhaustion and trauma-related responses (Bridgland et al., 2021). Accordingly, growing evidence indicates that the pandemic generated multiple overlapping stressors–including fear of infection, social isolation, uncertainty, and economic hardship–which have been associated with elevated levels of PTSD, particularly among vulnerable groups such as migrants (Saad et al., 2022). Furthermore, previous studies conducted during the pandemic have shown that events perceived as direct threats to life are more likely to elicit intense fear and uncertainty, key mechanisms underlying trauma-related symptomatology (Aragona et al., 2022).

### 4.4 Quality of life

The results also show significant negative associations between the mental component of QoL (MCS-12) and mental health outcomes, indicating that higher mental quality of life is associated with lower levels of PTSD, anxiety, and depression. These results are consistent with previous research demonstrating that a wide range of mental health disorders–including PTSD, anxiety, and depression–are strongly associated with poorer QoL (Eslam Parast and Taştekin Ouyaba, 2022; Van der Boor et al., 2020). In particular, Rapaport et al. (2005) reported that individuals with major depressive disorder, chronic depression, and PTSD exhibited some of the most severe impairments in QoL compared with population norms. Similarly, population-based studies have consistently shown that depressive and anxiety disorders are associated with substantial reductions in health-related QoL (Saarni et al., 2007).

Importantly, the existing literature also suggests that the relationship between mental health symptoms and QoL is likely bidirectional. While some longitudinal evidence indicates that lower QoL may predict subsequent increases in depressive symptoms (Hohls et al., 2021), other studies have also shown that greater symptom severity in depressive and anxiety disorders is associated with worse perceived QoL (Rapaport et al., 2005). However, given the cross-sectional design of the present study, QoL should be understood as an associated explanatory domain included within the analytical framework, rather than a unidirectional predictor. Therefore, the findings should be interpreted as reflecting cross-sectional associations rather than causal effects.

Additionally, the physical component (PCS-12) was negatively associated with anxiety and depression, highlighting the close relationship between physical and mental health. This finding aligns with previous evidence showing that anxiety and depression negatively impact both mental and physical dimensions of QoL, although their impact is generally greater on the mental component (Kuehner and Huffziger, 2009; Rapaport et al., 2005). Overall, these findings underscore the importance of considering QoL as a multidimensional construct closely intertwined with mental health among migrant individuals.

### 4.5 Strengths and limitations

The present study has several notable strengths that enhance the validity and relevance of its findings. It addresses an important and relatively understudied public health issue–the mental health of migrants in Spain– thereby contributing context-specific evidence to the broader literature. The relatively large and heterogeneous sample of 509 participants provided sufficient statistical power while capturing a broad range of migration backgrounds, offering a culturally nuanced perspective.

Methodologically, the use of standardized and validated instruments (CIDI, GAD-7, PCL-5 and SF-12) ensures rigor and facilitates comparability with previous studies. Robust analytical methods (Tobit and logistic regression models) along with adherence to ethical standards further strengthen the reliability of the findings. Moreover, the inclusion of a broad range of sociodemographic and psychosocial, migration-related, and sociocontextual factors grounded in a theoretically informed framework advances a multifactorial understanding of migrant mental health and has potential implications for policy and intervention.

Nonetheless, several limitations should be considered. First, the absence of a non-migrant comparison group means that the reported associations apply only to the migrant population studied and do not allow conclusions about differences between migrants and non-migrants or about whether the observed associations are specific to migrant populations. Consequently, potential intersectional effects between migration status and general risk factors (e.g., gender, age, and general stress) could not be disentangled. The use of convenience and snowball sampling may limit the sample’s representativeness and restrict generalizability. Participants with limited digital access or literacy; and those in more precarious legal or administrative situations who may be less likely to engage with online surveys; those experiencing greater residential instability or social isolation may have been underrepresented. As these groups are likely to experience greater vulnerability, the overall mental health burden among migrants in Spain may have been underestimated. The unequal gender distribution, with women representing 62.7% of the sample, may also have influenced prevalence estimates and the statistical power to detect gender-related associations, thereby limiting the generalizability of the findings. The inclusion of participants from many countries also required aggregation at the continental level for analytical purposes, which potentially masks important within-continent differences in cultural, socioeconomic, and migration-related characteristics. In addition, some subgroups–particularly participants from Europe (*n* = 6)–were very small. Although the association between European origin and PTSD remained in the same direction in sensitivity analyses, this finding should be interpreted with considerable caution and confirmed in larger studies.

Depression was assessed using a dichotomous diagnostic measure (CIDI), whereas anxiety and PTSD were analyzed as continuous symptom severity measures, potentially limiting comparability across outcomes. Self-reported measures are also subject to recall and social desirability biases. Furthermore, the cross-sectional design precludes establishing the directionality or causality of the observed associations. This is particularly relevant to the relationship between QoL and mental health outcomes, which is likely bidirectional. Finally, future longitudinal and comparative studies are needed to better understand temporal relationships between migration-related factors, QoL, and mental health outcomes, and to determine whether the observed associations differ from those in non-migrant populations.

## 5 Conclusion

This study provides new evidence on the interplay of factors associated with mental health among migrants residing in Spain. Our findings support a multifactorial framework in which sociodemographic, psychological, migration-related, post-migration social and occupational, and health-related QoL factors are associated with symptoms of PTSD, anxiety, and depression.

Although several of the identified factors, including gender, age, and childhood trauma, are well-established factors of mental health in the general population, our results indicate that they remain relevant among migrants and may interact with migration-related stressors to increase vulnerability. In addition, migration-related experiences, particularly acculturative stress and perceived discrimination, emerged as potentially modifiable factors, highlighting important opportunities for culturally adapted prevention and intervention strategies. These findings also emphasize that promoting migrant mental health requires approaches beyond the individual level, including structural barriers such as discrimination, while recognizing the broader social and environmental context in which migration takes place.

The consistent associations of childhood trauma, general stress, and the mental component of QoL with all three mental health outcomes underscore the impact of pre-migration adversities and ongoing psychosocial stressors, as well as the central role of subjective well-being in psychological adjustment related to migration. Together, these findings support the implementation of trauma-informed, culturally responsive, and community-based mental health services that address the multiple challenges experienced throughout the migration process. They also suggest that improving QoL should be a key component of mental health promotion and that integrated approaches may be particularly beneficial. Future longitudinal studies are needed to clarify the temporal relationships among these factors and to better understand the mechanisms through which migration-related experiences influence mental health over time. Such evidence will be essential for informing policies and interventions aimed at reducing mental health inequalities and promoting the long-term well-being and social inclusion of migrant populations in Spain.

## Data Availability

The raw data supporting the conclusions of this article are available on request to the corresponding author.
