# Supplementary material for: A multifactorial model of factors associated with PTSD, anxiety and depression among migrants in Spain
Source: Front Psychol. 2026 Aug 26;17:1848469. doi: 10.3389/fpsyg.2026.1848469 (PMC13553878; doi:10.3389/fpsyg.2026.1848469)
Supplement: Supplementary file 1 [file Table_1.docx]

Supplementary Material

Table S1. Assessment of multicollinearity using generalized variance inflation factors (GVIFs) for predictors included in the multivariable regression models.

|  | **PTSD** | | | **Anxiety** | | | **Depression** | | |
| --- | --- | --- | --- | --- | --- | --- | --- | --- | --- |
|  | GVIF | DF | GVIF^[1/2(2*DF) | GVIF | DF | GVIF^[1/2(2*DF) | GVIF | DF | GVIF^[1/2(2*DF) |
| **Sociodemographic and psychological characteristics** | | | | | | | | | |
| Gender | 1.353 | 2 | 1.078 | 1.336 | 2 | 1.075 | 1.540 | 2 | 1.114 |
| Age | 1.385 | 1 | 1.177 | 1.370 | 1 | 1.170 | 1.489 | 1 | 1.220 |
| Continent of origin | 2.787 | 3 | 1.186 | 2.737 | 3 | 1.182 | 3.017 | 3 | 1.202 |
| Civil status | 1.302 | 1 | 1.141 | 1.297 | 1 | 1.139 | 1.338 | 1 | 1.157 |
| Self-esteem | 1.583 | 1 | 1.258 | 1.604 | 1 | 1.266 | 1.605 | 1 | 1.267 |
| Resilience | 1.175 | 1 | 1.083 | 1.178 | 1 | 1.085 | 1.245 | 1 | 1.116 |
| Childhood trauma | 1.333 | 1 | 1.154 | 1.338 | 1 | 1.157 | 1.297 | 1 | 1.139 |
| **Migratory factors per se** | | | | | | | | | |
| Type of migration | 1.345 | 1 | 1.160 | 1.338 | 1 | 1.157 | 1.457 | 1 | 1.207 |
| Acculturative stress | 1.451 | 3 | 1.064 | 1.446 | 3 | 1.063 | 1.731 | 3 | 1.096 |
| Intention to remain in Spain | 1.190 | 1 | 1.091 | 1.190 | 1 | 1.091 | 1.195 | 1 | 1.093 |
| Residence status | 1.654 | 1 | 1.286 | 1.650 | 1 | 1.285 | 1.696 | 1 | 1.302 |
| Current Nationallity | 1.196 | 1 | 1.093 | 1.199 | 1 | 1.095 | 1.232 | 1 | 1.110 |
| Traveling companion status | 1.246 | 1 | 1.116 | 1.251 | 1 | 1.118 | 1.349 | 1 | 1.162 |
| Length of stay in Spain | 1.666 | 1 | 1.291 | 1.683 | 1 | 1.297 | 1.803 | 1 | 1.343 |
| **Social and occupational environment factors** | | | | | | | | | |
| Perceived discrimination | 1.793 | 1 | 1.339 | 1.770 | 1 | 1.331 | 1.831 | 1 | 1.353 |
| Feeling of inferiority | 1.391 | 1 | 1.180 | 1.378 | 1 | 1.174 | 1.442 | 1 | 1.201 |
| Social Support | 1.242 | 1 | 1.115 | 1.244 | 1 | 1.115 | 1.339 | 1 | 1.157 |
| Stressful life events | 1.372 | 1 | 1.171 | 1.364 | 1 | 1.168 | 1.370 | 1 | 1.170 |
| Current employment contract | 1.329 | 1 | 1.153 | 1.341 | 1 | 1.158 | 1.378 | 1 | 1.174 |
| Pandemic related factors | | | | | | | | | |
| Residence during the COVID-19 pandemic | 1.434 | 2 | 1.094 | 1.440 | 2 | 1.096 | 1.450 | 2 | 1.097 |
| COVID-19 diagnosis | 1.229 | 2 | 1.053 | 1.229 | 2 | 1.053 | 1.251 | 2 | 1.058 |
| COVID-related stress | 1.957 | 1 | 1.399 | 1.964 | 1 | 1.401 | 2.015 | 1 | 1.420 |
| General stress | 1.951 | 1 | 1.397 | 1.969 | 1 | 1.404 | 1.854 | 1 | 1.361 |
| Time since the study onset | 1.540 | 1 | 1.240 | 1.532 | 1 | 1.238 | 1.603 | 1 | 1.267 |
| **Health-related quality of life** | | | | | | | | | |
| Mental component score | 1.601 | 1 | 1.265 | 1.598 | 1 | 1.264 | 1.665 | 1 | 1.290 |
| Physical component score | 1.295 | 1 | 1.138 | 1.297 | 1 | 1.139 | 1.555 | 1 | 1.247 |

## Table S2. Sensitivity analysis of the association between European origin and PTSD symptoms

|  | | **PTSD** | | **Anxiety** | | **Depression** | |
| --- | --- | --- | --- | --- | --- | --- | --- |
|  | | Β (SE) | p-value | Β (SE) | p-value | OR (SE) | p-value |
| **Sociodemographic and psychological characteristics** | | | | | | | |
| Gender | Male | Ref. | | Ref. | | Ref. | |
|  | Female | 0.22 (0.32) | 0.500 | **1.20 (0.45)** | **0.008** | **1.07 (0.32)** | **<0.001** |
|  | Other (transgender, agender, gender fluid) | **-4.04 (1.91)** | **0.034** | 1.44 (1.39) | 0.300 | -0.20 (1.12) | 0.859 |
| Age | | -0.01 (0.02) | 0.604 | **-0.04 (0.02)** | **0.036** | -0.00 (0.01) | 0.781 |
| Continent of origin | No-Europe | Ref. | | Ref. | | Ref. | |
|  | Europe | **2.73 (1.21)** | **0.024** | 0.73(1.65) | 0.66 | 0.95 (0.99) | 0.340 |
| Civil status | In a relationship | Ref. | | Ref. | | Ref. | |
|  | Single | 0.42 (0.33) | 0.212 | -0.14 (0.45) | 0.747 | -0.16 (0.31) | 0.593 |
| Self-esteem | | -0.01 (0.04) | 0.782 | 0.04 (0.05) | 0.427 | 0.02 (0.03) | 0.548 |
| Resilience | | 0.00 (0.17) | 0.980 | 0.15 (0.21) | 0.482 | 0.23 (0.15) | 0.125 |
| Childhood trauma | | **0.04 (0.01)** | **<0.001** | **0.03 (0.01)** | **0.023** | **0.02 (0.01)** | **0.018** |
| **Migratory factors per se** | | | | | | | |
| Type of migration | Voluntary | Ref. | | Ref. | | Ref. | |
|  | Forced | 0.12 (0.37) | 0.741 | 0.55 (0.47) | 0.246 | 0.33 (0.32) | 0.312 |
| Acculturative stress | Never | Ref. | | Ref. | | Ref. | |
|  | Occasionally | 0.59 (0.40) | 0.145 | 0.13 (0.55) | 0.821 | -0.49 (0.35) | 0.164 |
|  | Often | 0.61 (0.60) | 0.306 | 0.47 (0.89) | 0.600 | -1.04 (0.57) | 0.067 |
|  | Very often | 1.04 (1.11) | 0.345 | **4.29 (1.50)** | **0.004** | 1.21 (0.94) | 0.198 |
| Intention to remain in Spain | Uncertain | Ref. | | Ref. | | Ref. | |
|  | Intention to stay | -0.12 (0.32) | 0.719 | 0.51 (0.42) | 0.233 | 0.23 (0.28) | 0.415 |
| Residence status | Undocumented or in process | Ref. | | Ref. | | Ref. | |
|  | Residence permit | 0.13 (0.39) | 0.745 | -0.61 (0.47) | 0.213 | -0.37 (0.32) | 0.237 |
| Travelling companion status | Accompanied | Ref. | | Ref. | | Ref. | |
|  | Alone | 0.44 (0.30) | 0.145 | 0.15 (0.42) | 0.714 | **0.62 (0.30)** | **0.041** |
| Length of stay in Spain | | -0.00 (0.02) | 0.883 | 0.03 (0.03) | 0.397 | -0.03 (0.02) | 0.256 |
| **Social and occupational environment factors** | | | | | | | |
| Perceived discrimination | | **0.15 (0.04)** | **<0.001** | **0.13 (0.06)** | **0.029** | 1.00 (0.04) | 0.951 |
| Feeling of inferiority | No | Ref. | | Ref. | | Ref. | |
|  | Yes | -0.21 (0.34) | 0.533 | 0.62 (0.45) | 0.868 | 0.08 (0.31) | 0.802 |
| Social support | | 0.13 (0.15) | 0.391 | -0.15 (0.21) | 0.942 | -0.11 (0.14) | 0.450 |
| Stressful life events | | 0.10 (0.05) | 0.096 | 0.05 (0.07) | 0.486 | 0.02 (0.04) | 0.548 |
| Employment contract | No | Ref. | | Ref. | | Ref. | |
|  | Yes | -0.45 (0.41) | 0.271 | -0.08 (0.51) | 0.867 | 0.13 (0.35) | 0.715 |
| Pandemic-related factors | | | | | | | |
| Residence during the COVID-19 pandemic | Yes, during the entire period | Ref. | | Ref. | | Ref. | |
|  | Yes, but also in other countries | 0.15 (0.78) | 0.849 | 1.51 (0.92) | 0.100 | -0.02 (0.68) | 0.980 |
|  | No | -0.33 (0.50) | 0.502 | 0.57 (0.65) | 0.378 | -0.72 (0.54) | 0.180 |
| COVID-19 diagnosis | Yes | Ref. | | Ref. | | Ref. | |
|  | Yes and hospitalization | -0.07 (1.16) | 0.951 | 1.59 (1.62) | 0.329 | -0.14 (1.11) | 0.900 |
|  | No | 0.20 (0.35) | 0.563 | -0.47 (0.49) | 0.335 | -0.33 (0.32) | 0.309 |
| COVID-related stress | | **0.17 (0.04)** | **<0.001** | 0.07 (0.06) | 0.224 | **-0.10 (0.03)** | **0.003** |
| General stress | | **0.10 (0.03)** | **0.003** | **0.10 (0.04)** | **0.019** | **0.07 (0.03)** | **0.005** |
| Time since study onset | | 0.00 (0.00) | 0.380 | 0.00 (0.00) | 0.768 | 0.00 (0.00) | 0.318 |
| **Health-related quality of life** | | | | | | | |
| Mental component score | | **-0.10 (0.02)** | **<0.001** | **-0.28 (0.02)** | **<0.001** | **-0.11 (0.02)** | **<0.001** |
| Physical component score | | -0.03 (0.02) | 0.170 | **-0.12 (0.02)** | **<0.001** | **-0.05 (0.01)** | **0.003** |
